# Clinical burden of major depressive disorder with versus without prominent anhedonia using a real-world electronic health records and claims linked database

**DOI:** 10.1186/s12888-025-07139-x

**Published:** 2025-07-25

**Authors:** Hrishikesh Kale, Stevan Geoffrey Severtson, Becca S. Feldman, Tiina Drissen, Nilanjana Dwibedi, Andrew J. Cutler, Carl D. Marci

**Affiliations:** 1 Johnson & Johnson, Titusville, NJ USA; 2 OM1, Inc., Boston, MA USA; 3 SUNY Upstate Medical University, Lakewood Ranch, FL USA; 4 Massachusetts General Hospital, Harvard Medical School, Boston, MA USA

**Keywords:** Anhedonia, Major depressive disorder, PHQ-9, Treatment patterns, Antidepressants

## Abstract

**Background:**

Anhedonia is a core feature of major depressive disorder (MDD), yet the clinical burden in real-world settings is not well understood. This retrospective cohort study assessed depression symptoms and treatment patterns among patients with MDD with and without prominent anhedonia.

**Methods:**

Patients with MDD were selected from a real-world dataset of electronic health records from mental health specialists and linked medical and pharmacy claims (OM1, Inc. Boston MA) between January 2013 through August 2023. Patients were included if the first Patient Health Questionnaire 9-item (PHQ-9) score indicated moderate to severe depression (≥ 10) with a MDD diagnosis +/-30 days. The date of the patients’ first PHQ-9 score was used as the index date. Patients with a score ≥ 2 on PHQ-9 item 1 (anhedonia) at the index date were classified as MDD with prominent anhedonia (MDD-ANH) (*n* = 4,255). The remaining patients were classified as Other-MDD (*n* = 1,454). Treatment patterns were assessed using prescription records in the year following the index date. The last PHQ-9 score in the year following the index date was used to assess remission rates (PHQ-9 score < 5) or persistence of moderate to severe depression.

**Results:**

A total of 5,709 patients with MDD were assessed; 74.5% were in MDD-ANH and 25.5% were in the Other-MDD cohort (met criteria for MDD but not prominent anhedonia). The mean index PHQ-9 score was 18.2 (SD = 4.2) for MDD-ANH patients and 13.5 (SD = 2.9) for Other-MDD patients. The percentage of patients treated with antidepressants was 86.4% in the MDD-ANH group and 84.7% in the Other-MDD group. After adjusting for baseline characteristics, patients with MDD-ANH were more likely to have been treated with atypical antipsychotics (OR = 1.51, *p* < 0.001), more likely to have switched medication (OR = 1.24, *p* = 0.004), and more likely to have augmented antidepressant therapy (OR = 1.16, *p* = 0.021) than Other-MDD patients. Patients with MDD-ANH were less likely to have achieved remission (OR = 0.82, *p* = 0.003) and were more likely to have persistent depression (OR = 1.50, *p* < 0.001) than patients with Other-MDD.

**Conclusions:**

Overall, MDD-ANH patients had higher clinical burden reflected in more depressive symptoms, higher treatment utilization and lower rates of remission compared to Other-MDD.

**Supplementary Information:**

The online version contains supplementary material available at 10.1186/s12888-025-07139-x.

## Introduction

Major depressive disorder (MDD) is a common psychiatric disease in the United States with an estimated 20% lifetime prevalence among adults [1]. Anhedonia, defined as “markedly diminished interest or pleasure in all, or almost all activities most of the day” [2], is one of the diagnostic criteria for MDD. The presence of anhedonia in MDD is associated with failure to respond adequately to treatment and other adverse outcomes [3, 4]. Inadequate response to MDD treatment is associated with increased health care utilization and costs [5, 6].

Estimates of anhedonia prevalence among patients with MDD in clinical trials and cross-sectional studies have varied substantially, ranging from 40 to 70% when measured using patient-reported questionnaires [7, 8]. Higher levels of anhedonia in these types of studies are associated with lower self-reported health-related quality of life [9]. The presence of anhedonia is also correlated with greater severity of depression symptoms [10, 11] and remains a common complaint by patients with MDD after initiating antidepressant treatment [12]. Anhedonia may also be associated with inflammation in depression, which may contribute to poorer outcomes in patients with MDD [13]. Anhedonia has been linked with increased suicidal ideation [14]. Given the prevalence and association with poor outcomes, adequate treatment for anhedonia could help reduce the high burden of MDD with anhedonia.

Though anhedonia is a core symptom of depression and associated with adverse outcomes, the presence and outcomes among patients in clinical, real-world settings is not well understood and a search of the literature found no published studies using real-world clinical data sources. A limiting factor may be the capture rates of specific symptoms in EHR or claims data sources and limited use of questionnaires assessing anhedonia in clinical practice. The Patient Health Questionnaire-9 (PHQ-9) [15, 16] is a reliable and well-validated questionnaire frequently used to screen patients for depression in clinical settings. The PHQ-9 provides information on the frequency that patients experience specific depressive symptoms, including anhedonia. The aim of this study was to examine the clinical burden of anhedonia, assessed using the first item from the PHQ-9 in the real-world. While doing so the study assessed the association between elevated anhedonia scores with treatment patterns and remission scores in patients with MDD. The study used information collected from EHR data from patients seeking treatment in a network of mental health clinicians with linked medical and pharmacy claims to compare the clinical burden reflected in treatment patterns and the persistence of depressive symptoms among adult patients with MDD with prominent anhedonia (MDD-ANH) to adults with MDD with no or low levels of anhedonia (Other-MDD).

## Materials and methods

### Study design and sample

A retrospective observational cohort study of adult patients with MDD was conducted using data from the OM1 PremiOM™ MDD Dataset, a continuously updated, deterministically linked, multi-source dataset derived from EHR and claims data. (OM1, Inc. Boston MA). The medical and pharmacy claims contain billing and coding history on inpatient and outpatient encounters from acute care facilities, ambulatory medical and surgery centers, and a variety of specialty clinics. The EHR data comes from the OM1 Mental Health Specialty Network. The OM1 Mental Health Specialty Network includes over 3.5 million patients seen by more than 9,000 specialists in over 2,500 community-based practices in all 50 states and includes clinical data within both structured and unstructured EHR fields, including patient-reported measures such as the PHQ-9. Information is obtained from patient encounters from specialty providers in both outpatient and inpatient settings. More than 95% percent of clinical health care encounters used in these analyses were from outpatient encounters.

The PHQ-9 was used to identify patients with MDD who had moderate to severe depression, defined as a PHQ-9 total score of ≥ 10 at baseline. The PHQ-9 is composed of nine items assessing symptoms of depression. For each item, respondents are asked how often they have been bothered by the symptoms listed over the two weeks prior to administration of the questionnaire. There are four potential responses for each item: 0=”not at all,” 1=”several days,” 2=”more than half the days,” or 3=”nearly every day.” Items are summed with total scores ranging from 0 to 27. The index date was the date of the first complete PHQ-9 score for each patient with 12 months of linked claims and EHR data before and after the assessment. Moderate to severe depression was confirmed by diagnosis of active MDD within 30 days pre- or post-index date. To approximate a new major depressive episode, patients with diagnoses indicating active or remitted MDD prior to the 30 days before initial PHQ-9 assessment and within one year of the PHQ-9 assessment were excluded.

Patients with other commonly cooccurring psychiatric disorder diagnoses, such as anxiety or attention deficit-hyperactivity disorders, were retained to reflect patients treated for major depressive disorder in real-world settings. However, we wanted to isolate the effects of anhedonia that was a symptom of major depressive disorder. Therefore, patients with bipolar disorders were excluded to ensure that we were evaluating patients with unipolar depression. We excluded patients with schizophrenia and schizoaffective disorders because we wanted to assess anhedonia as a symptom of major depression. Finally, patients with substance use disorders were excluded to minimize depressive symptoms that may be attributed to misuse of alcohol or other drugs.

## Measures

### Prominent anhedonia definition

The first question (Item 1) of the PHQ-9 [15] was used to define two cohorts. MDD with prominent anhedonia (MDD-ANH) was defined as a score of ≥ 2 on Item 1 which evaluates the degree of ”Little interest or pleasure in doing things.” A score of 2 or greater indicates experiencing this symptom more than half the days or nearly every day. This threshold was selected because it is consistent with the symptom frequency for diagnosis of a major depressive episode based on the DSM-5 [2]. Other-MDD patients were moderately to severely depressed patients at the index date with a score < 2 on Item 1. The baseline period was the 12 months prior to the index date. The follow-up period was the 12 months post-index date (including the index date).

### Demographic and comorbidity information

Demographic information including age, sex, race, and ethnicity information was extracted from patients’ EHR records in the OM1 PremiOM MDD Dataset. Information on insurance status and comorbidities were extracted from EHR or medical claims. Insurance represents use of any type of insurance for a medical encounter in the year prior to the index date. Medical comorbidities are assessed using the Charlson Comorbidity Index, a score based on the number of chronic illnesses a patient is experiencing [17]. The Charlson Comorbidity scores are based on a standardized set of diagnoses provided any time prior to the index date [17, 18]. Scores range from 0 to 37 with higher scores associated with higher risk of mortality [17]. Attention deficit hyperactivity disorder (ADHD), any anxiety disorder, and post-traumatic stress disorder (PTSD) were psychiatric comorbidities assessed in the year prior to the index date.

### PHQ-9 total scores

The latest available PHQ-9 score after index was used to assess change in depressive symptoms. PHQ-9 scores where all individual items were documented in structured EHR notes in the OM1 PremiOM MDD Dataset were used. All scores during follow-up were between six months and one year after the index date.

### Remission

Remission was defined as patients who achieved a PHQ-9 score of < 5 which is a threshold used across other studies [19–21]. Remission was calculated based on the latest PHQ-9 score during follow-up in the MDD-ANH and Other-MDD cohorts.

### Persistent depression

Persistent depression was defined as patients whose PHQ-9 score remained 10 or greater. Persistent depression was calculated based on the latest PHQ-9 score during follow-up in the MDD-ANH and Other-MDD cohorts.

### Antidepressant treatment

Information on medication usage was obtained from prescription fills from pharmacy claims or medication orders from EHR records. Antidepressants treatments in the follow-up period were presented among patients from the MDD-ANH cohort and the Other-MDD cohorts. The percentage of patients receiving antidepressants of adequate duration (≥ 30 days of treatment with no more than 14-day gap) are presented. Duration was determined by adding the day’s supply to the prescription fill or medication order date. Antidepressants included were categorized as selective serotonin reuptake inhibitors (SSRIs), serotonin and norepinephrine reuptake inhibitors (SNRIs), tricyclic antidepressants, atypical antidepressants, and monoamine oxidase inhibitors (MAOIs).

### Switching antidepressants

Switching to another line of antidepressant therapy was defined as starting a unique antidepressant therapy after at least 30 days of continuous treatment on the previous line of therapy and where the original line of therapy ended within 30 days of the start of the next antidepressant.

### Augmentation

Augmentation was defined as the addition of another antidepressant or non-antidepressant therapy to a given line of therapy. Non-antidepressants considered for augmentation are provided in supplemental Table 1. Augmentation was considered if the start date of the augmenting therapy was after at least 30 days of continuous treatment with the original treatment and the second antidepressant was of adequate duration (≥ 30 days with no gaps greater than 14 days). The use of non-antidepressant augmentation medication could be of any duration. The prescription duration of the original antidepressant and the second antidepressant had to overlap at least 30 days to be considered augmentation. If the overlap was less than 30 days, it was considered a switch.

### Statistical analysis

Descriptive statistics were used for demographic variables. The presence or absence of any antidepressant treatment, any individual psychotherapy treatment, any antidepressant switch, and any antidepressant augmentation was assessed. In addition, any treatment with SSRIs, SNRIs, tricyclic antidepressants, atypical antidepressants, mood stabilizers, or atypical antipsychotic was assessed. Finally, presence or absence of persistent depression or remission at last available PHQ-9 assessment during follow-up was analyzed. These binary outcomes were assessed using bivariate and multivariable logistic regression models. Unadjusted and adjusted odds ratios are presented for MDD-ANH relative to the Other-MDD cohort. Adjusted analysis controlled for age at baseline, insurance status, attention-deficit/hyperactivity disorder (ADHD) diagnosis any time prior to baseline, and Charlson Comorbidity Index score.

## Results

### Study sample

A total of 5,709 patients with MDD were assessed; 74.5% were in MDD-ANH and 25.5% were in the Other-MDD cohort (met criteria for MDD but not prominent anhedonia). Patient demographics and baseline characteristics during the 12-month baseline period are displayed in Table 1 for each of the two cohorts. The mean (SD) age in years was greater in the MDD-ANH (41.0, [SD = 15.8]) than the Other-MDD (37.8, [15.0], *p* < 0.001). A greater percentage of MDD-ANH patients had used Medicare (9.0% for MDD-ANH to 5.5% for Other-MDD, *p* < 0.001) or Medicaid (7.4% for MDD-ANH vs. 5.7% for Other-MDD, *p* = 0.033) for their most recent health care encounter prior to index. MDD-ANH patients tended to have more non-psychiatric baseline comorbidities compared to Other-MDD at any point prior to baseline. Mean (SD) of Charlson Comorbidity Index scores were greater among the MDD-ANH patients (0.6 [1.3]) compared to Other-MDD patients (0.5 [1.1], *p* = 0.020). There were no statistically significant differences in baseline psychiatric comorbidities between the MDD-ANH and Other-MDD groups, though the proportion of patients with ADHD was slightly lower in MDD-ANH (9.7%) compared to Other-MDD (11.3%).

**Table 1:** Demographic information by cohort

Variable	Characteristic	MDD-ANH (*N* = 4,255)	Other-MDD (*N* = 1,454)	*p*-value
Age (years)	Mean (SD)	41.0 (15.8)	37.8 (15.0)	< 0.001
Sex	Female	3,065 (72.0%)	1,077 (74.1%)	0.133
	Male	1,190 (28.0%)	377 (25.9%)	
Race	Asian	29 (1.4%)	8 (1.1%)	0.468
	Black/AA	159 (7.5%)	47 (6.2%)	
	Caucasian	1,834 (87.0%)	664 (87.9%)	
	Other (including multiple)	85 (4.0%)	36 (4.8%)	
	Unknown/Not Reported	2,148	699	
Ethnicity	Hispanic/Latino	203 (10.0%)	82 (11.3%)	0.319
	Non-Hispanic/Latino	1,822 (90.0%)	641 (88.7%)	
	Unknown/Not Reported	2,230	731	
Insurance: Commercial	N (%)	2,014 (47.3%)	718 (49.4%)	0.177
Insurance: Medicare	N (%)	384 (9.0%)	80 (5.5%)	< 0.001
Insurance: Medicaid	N (%)	313 (7.4%)	83 (5.7%)	0.033
Comorbid Anxiety Disorder	N (%)	1,915 (45.0%)	656 (45.1%)	0.941
Comorbid ADHD	N (%)	413 (9.7%)	164 (11.3%)	0.086
Comorbid Post-Traumatic Stress Disorder	N (%)	286 (6.7%)	89 (6.1%)	0.425
Charlson Comorbidity Index	Mean (SD)	0.6 (1.3)	0.5 (1.1)	0.020
Baseline PHQ-9 score	Mean (SD)	18.2 (4.2)	13.5 (2.9)	< 0.001

*P*-values for categorical variables were calculated using a chi-square test, *p*-values for continuous variables are calculated using the Mann-Whitney U test

The mean PHQ-9 score at the index date was higher among patients with MDD-ANH (18.2, SD = 4.2) compared to patients with Other-MDD (13.5, SD = 2.9) (Table 1). In addition, the last PHQ-9 score during follow-up was higher among patients with MDD-ANH (9.4, SD = 6.6) compared to patients with Other-MDD (8.1, SD = 5.8) (Table 1).

Analysis of antidepressant treatment patterns is presented in Table 2. In both cohorts, more than 80% of patients were treated with antidepressants and individual psychotherapy during follow-up. In the multivariable analyses, patients with MDD-ANH were more likely to have switched antidepressants (adjusted odds ratio [aOR] = 1.24, 95% CI: 1.07, 1.44, *p* = 0.004) and were more likely to have augmented antidepressant therapy (aOR = 1.16, 95% CI: 1.02, 1.31, *p* = 0.021) compared to the Other-MDD group.

The percentage of patients using different pharmacologic therapies at follow-up is presented in Table 2. After adjustment for baseline characteristics, patients with MDD-ANH were more likely to have been treated with SNRIs (aOR = 1.29, 95% CI: 1.13, 1.47, *p* < 0.001), tricyclic antidepressants (aOR = 1.31, 95% CI: 1.10, 1.56, *p* = 0.003), mood stabilizers (aOR = 1.32, 95% CI: 1.15, 1.51, *p* < 0.001), atypical antidepressants (aOR = 1.24, 95% CI: 1.09, 1.40, *p* < 0.001), and atypical antipsychotics (aOR = 1.51, 95% CI: 1.31, 1.74, *p* < 0.001) during the follow-up time period.

**Table 2:** Analyses of treatment patterns and medication use in the 12 month follow up period by cohort

Outcome Variable	MDD-ANH (*N* = 4,255) *N* (%)	Other-MDD (*N* = 1,454) *N* (%)	Unadjusted odds ratio (95% CI)	Unadjusted odds ratio *p*-value	Adjusted odds ratio (95% CI)	Adjusted odds ratio *p*-value
**Treatment Patterns**						
Any antidepressant N (%)	3,677 (86.4%)	1,231 (84.7%)	1.15 (0.97, 1.36)	0.097	1.14 (0.96, 1.35)	0.123
Psychotherapy N (%)	3,681 (86.5%)	1,274 (87.6%)	0.91 (0.76, 1.08)	0.280	0.90 (0.75, 1.08)	0.252
Antidepressant switch N (%)	975 (22.9%)	278 (19.1%)	1.26 (1.08, 1.46)	0.003	1.24 (1.07, 1.44)	0.004
Augmentations N (%)	1,765 (41.5%)	546 (37.6%)	1.18 (1.04, 1.33)	0.008	1.16 (1.02, 1.31)	0.021
**Medication Use**						
SSRIs N (%)	2,491 (58.5%)	891 (61.3%)	0.89 (0.79, 1.01)	0.067	0.93 (0.83, 1.06)	0.283
SNRIs N (%)	1,514 (35.6%)	425 (29.2%)	1.34 (1.18, 1.52)	< 0.001	1.29 (1.13, 1.47)	< 0.001
Tricyclic antidepressants N (%)	736 (17.3%)	186 (12.8%)	1.43 (1.20, 1.70)	< 0.001	1.31 (1.10, 1.56)	0.003
Atypical antidepressants N (%)	1,754 (41.2%)	524 (36.0%)	1.24 (1.10, 1.41)	< 0.001	1.24 (1.09, 1.40)	< 0.001
Mood stabilizers N (%)	1,271 (29.9%)	352 (24.2%)	1.33 (1.16, 1.53)	< 0.001	1.32 (1.15, 1.51)	< 0.001
Atypical antipsychotics N (%)	1,308 (30.7%)	325 (22.4%)	1.54 (1.34, 1.77)	< 0.001	1.51 (1.31, 1.74)	< 0.001
**Last PHQ-9 During 12-Month Follow Up**						
N (%) Persistent Depression (PHQ-9 score ≥ 10)	1,897 (44.6%)	510 (35.1%)	1.49 (1.32–1.68)	< 0.001	1.50 (1.32–1.70)	< 0.001
N (%) in remission (PHQ-9 score < 5)	1,222 (28.7%)	473 (32.5%)	0.84 (0.74–0.95)	0.006	0.82 (0.72–0.94)	0.003

Unadjusted odds ratios are estimated from simple logistic regression models regressing the outcome on cohort. Adjusted odds ratios are estimated from multivariable logistic regression models regressing outcome on cohort, age, ADHD, Charlson comorbidity index score, and insurance status. For each logistic regression, the outcome variable is regressed on cohort (MDD-ANH or MDD-Other). Other MDD is the reference group for each odds ratio

**Fig. 1:**
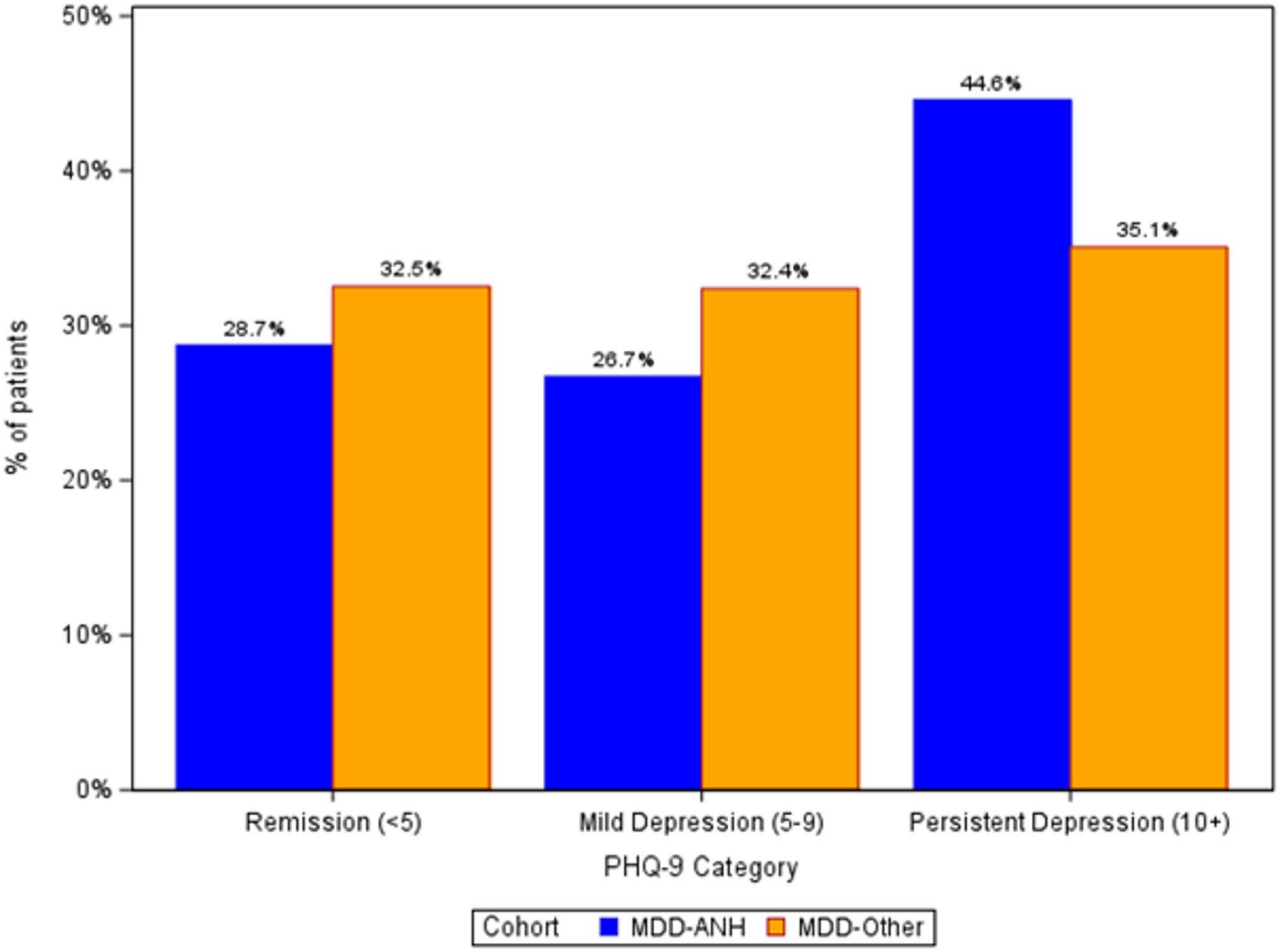
Percentage Of Respondents in Remission, with Mild Depression, or Persistent Depression at Latest PHQ-9 Assessment in the 12 Month Follow Up Period

The percentages of patients in remission (PHQ-9 score < 5), with mild depression (PHQ-9 score 5–9), and patients with persistent depression (PHQ-9 score ≥ 10) by cohort is displayed in Fig. 1. By the end of the 12-month follow-up period, 28.7% of patients in the MDD-ANH achieved remission compared with 32.5% of MDD-Other patients. Persistent moderate to severe depression was present among 44.6% of MDD-ANH patients by end of follow-up compared to 35.1% of MDD-Other patients. After adjustment for baseline characteristics, when compared to the Other-MDD group, patients in the MDD-ANH cohort were significantly more likely to have persistent depression (aOR = 1.50, 95% CI; 1.32, 1.70, *p* < 0.001) and were significantly less likely to be in remission by the final PHQ-9 assessment (aOR = 0.82, 95% CI: 0.72, 0.94, *p* = 0.003) (Table 2).

## Discussion

This real-world observational, retrospective cohort study assessed the clinical burden reflected in treatment patterns and persistence of depressive symptoms in patients with MDD with prominent anhedonia using EHR and linked claims data from a large network of mental health specialists within the US. Other studies have found anhedonia to be associated with poorer outcomes among patients with MDD [5, 6, 9–14]. The findings of this study are consistent in that patients with MDD with prominent anhedonia had greater MDD severity at baseline with lower remission rates and higher rates of persistence of depressive symptoms during follow-up. Patients with anhedonia were also more likely to be treated with atypical antipsychotics, more likely to have switched antidepressant medication, and were more likely to have augmented antidepressant therapy than Other-MDD patients.

In this real-world study, anhedonia was highly prevalent among patients with MDD. Of the 5,709 patients with MDD meeting inclusion criteria, 74.5% reported prominent anhedonia at baseline. This is higher than the upper range of published prevalence estimated of 40–70% [7, 8] but is consistent with findings from a small sample of patients with MDD meeting the threshold for anhedonia using the Snaith–Hamilton Pleasure Scale (SHAPS) [22, 23]. Reasons for the higher prevalence in this sample may include the fact that this is a treatment-seeking population that is more severely depressed than patients sampled in other studies. It may also be that patients in the real-world with a more severe form of MDD are more likely to be asked to complete the PHQ-9. Finally, the variations in prevalence estimates observed across studies may be due to challenges in measuring anhedonia. Rizvi and colleagues note that anhedonia is complex, multifaceted construct that may not be adequately captured using existing questionnaires [24]. This is likely relevant to this study which used a single item from the PHQ-9 to assess anhedonia. We are not aware of other studies that have used this approach to define anhedonia. These findings highlight the need for increased investigation into identifying reliable, valid, and brief assessments of anhedonia along with other symptoms of depression that can be applied in clinical settings.

The similarity in demographic and baseline characteristics between the MDD-ANH and Other-MDD cohorts was a notable finding. No differences were observed in sex, race, or ethnicity and few differences were observed in psychiatric comorbidities prior to the index date. The primary difference between cohorts was that MDD-ANH patients tended to be older. Other differences between the MDD-ANH and Other-MDD cohorts, such as differences in insurance type and non-psychiatric comorbidities as measured by the Charlson Comorbidity Index, may be attributed to the observed differences in age. This differs from another study that showed anhedonia was more prevalent among younger hospitalized patients with MDD [23]. These findings may be attributed to patient characteristics within the specialist network used to identify this cohort. Further investigation of this association is warranted.

Consistent with the differences observed in total scores, patients with MDD-ANH were less likely to experience remission and were more likely to have persistent depression. This finding is consistent with other studies that have observed more severe depressive symptoms among patients with anhedonia [10]. There was a large difference between the MDD-ANH and Other-MDD cohorts in the initial PHQ-9 scores, with MDD-ANH patients having higher scores compared to patients with Other-MDD. This finding was also observed during follow-up, though the magnitude of the difference between cohorts decreased. The difference in the magnitude of change from baseline to follow-up in PHQ-9 scores between the two cohorts may be related to the greater number of treatments received during follow-up by the MDD-ANH group. Patients with prominent anhedonia received more antidepressant medications and were more likely to be treated with atypical antipsychotics. Patients with MDD-ANH were also more likely to have more antidepressant switches and more augmentations of antidepressant treatments. These results suggest that prominent anhedonia at baseline is associated with a more complex treatment pattern and possible treatment failures. This study did not evaluate whether response to treatments differed by anhedonia severity at baseline. Future studies can investigate the potential for differential effectiveness in real-word populations.

There are several strengths to this study. Data are captured on all patients in a real-world setting, which captures the natural variations and deviations of treatments outside of traditional clinical trial settings. Inclusion of medical and pharmacy claims and EHR data provides a rich source of real-world clinical and administrative data to assess treatment patterns. The open-source nature of the claims means that patients can be followed for an extended period, for example, if they switch insurance providers due to changes in employment. The large data source used provided a diverse sample of all adult age groups and US census regions from which to identify patients. In addition, anhedonia and depressive symptoms were measured using a valid and reliable tool administered to thousands of patients in a real-world setting.

There are also notable limitations to this study. PHQ-9 scores in the dataset come from the EHR and were captured as part of their routine medical care. Therefore, variations exist in the availability of observed scores across patients. The use of retrospective data collected as part of routine clinical care limits the types of scales that could be used to assess depression and anhedonia to patient reported outcomes such as the PHQ-9 used in this study. Information on the duration or frequency of major depressive episodes was not examined as part of this study. The PHQ-9 is a self-report measure and subject to information biases such as inaccurate recall of symptoms. Another limitation is that this is a treatment-seeking sample of patients with MDD from a network of mental health providers. Therefore, findings are not generalizable to patients with MDD in the general population. Finally, due to the multi-source nature of the data, gaps may exist in a patient’s treatment history. These analyses examine patients whose first MDD is identified within one year of initial PHQ-9 assessment. While a proxy for an early depressive episode, the timing of treatment initiation may vary across patients.

## Conclusions

Overall, these findings highlight that patients with prominent anhedonia tend to have more severe depression; are less likely to achieve remission in follow-up; are more likely to have persistent depression; and require more antidepressant and augmentation treatments compared to patients without prominent anhedonia. These findings are consistent with other studies that have demonstrated that symptoms of anhedonia are associated with lower quality of life, risk of suicide, and impaired function. The greater association with poor outcomes highlights the importance of identifying and treating anhedonia in clinical practice. The development of effective treatments that target anhedonia could significantly improve clinical outcomes in a substantial number of patients with MDD.

## Supplementary Information

Supplementary Material 1.

## Data Availability

Restrictions apply to the availability of these data. The datasets generated and/or analyzed for the current study are not publicly available due to the confidential and proprietary nature of the datasets.
