# Supplementary material for: Clinical burden of major depressive disorder with versus without prominent anhedonia using a real-world electronic health records and claims linked database
Source: BMC Psychiatry. 2025 Jul 25;25:727. doi: 10.1186/s12888-025-07139-x (PMC12291255; doi:10.1186/s12888-025-07139-x)
Supplement: Supplementary file 1 — Supplementary Material 1. [file 12888_2025_7139_MOESM1_ESM.docx]

**Supplemental Table 1. Non-antidepressants considered for augmentation**

| **Category** | **Generic Name** |
| --- | --- |
| Second-generation (atypical) antipsychotics | Aripiprazole |
|  | Brexpiprazole |
|  | Cariprazine |
|  | Olanzapine |
|  | Quetiapine |
| Anticonvulsants | Lamotrigine |
|  | Carbamazepine |
|  | Gabapentin |
|  | Hypothyroidism medications |
|  | Valproate |
|  | Valproic acid |
|  | Oxcarbazepine |
|  | Topiramate |
| Other mood stabilizers | Lithium |
| Hypothyroidism medications | Desiccated thyroid |
|  | Liothyronine |
|  | Levothyroxine |
|  | Liotrix (combo) |
| Psychostimulants | Amphetamine |
|  | Dextroamphetamine |
|  | Lisdexamphetamine |
|  | Mixed amphetamine salts (Amphetamine and Dextroamphetamine) |
|  | Methylphenidate |
|  | Dexmethylphenidate |
| Non-benzodiazepine anxiolytics | Buspirone |
| Miscellaneous | Atomoxetine |
|  | Viloxazine |
| Oral benzodiazepine anxiolytics | Alprazolam |
|  | Chlordiazepoxide |
|  | Clonazepam |
|  | Clorazepate |
|  | Diazepam |
|  | Lorazepam |
|  | Oxazepam |
| Non-Benzodiazepine sleep agents | Eszopiclone |
|  | Zaleplon |
|  | Zolpidem |
| DORAS | Suvorexant |
|  | Lemborexant |
|  | Daridorexant |
